# Simulation of Laser-assisted Directed Energy Deposition of Aluminum Powder: Prediction of Geometry and Temperature Evolution

**DOI:** 10.3390/ma12132100

**Published:** 2019-06-29

**Authors:** Fabrizia Caiazzo, Vittorio Alfieri

**Affiliations:** Department of Industrial Engineering, University of Salerno, Via Giovanni Paolo II 132, 84084 Fisciano, Italy

**Keywords:** laser, directed energy deposition, aluminum, simulation

## Abstract

One of the main current challenges in the field of additive manufacturing and directed energy deposition of metals, is the need for simulation tools to prevent or reduce the need to adopt a trial-and-error approach to find the optimum processing conditions. A valuable help is offered by numerical simulation, although setting-up and validating a reliable model is challenging, due to many issues related to the laser source, the interaction with the feeding metal, the evolution of the material properties and the boundary conditions. Indeed, many attempts have been reported in the literature, although some issues are usually simplified or neglected. Therefore, this paper is aimed at building a comprehensive numerical model for the process of laser-assisted deposition. Namely: the geometry of the deposited metal is investigated in advance and the most effective reference shape is found to feed the simulation as a function of the governing factors for single- and multi-track, multi-layer deposition; then, a non-stationary thermal model is proposed and the underlying hypotheses to simulate the addition of metal are discussed step-by-step. Validation is eventually conducted, based on experimental evidence. Aluminum alloy 2024 is chosen as feeding metal and substrate.

## 1. Introduction

In the frame of additive manufacturing, the process of directed energy deposition (DED) is effective and minimally invasive; therefore it is receiving increasing interest in industry to prevent replacement of price-sensitive metal products [1,2]. The technology is based on a laser beam scanning the surface and creating a melting pool over an existing substrate; then, metal in the form of powder or wire is fed concurrently, in a single stage process. As a result, additional metal can be applied over worn-out surfaces, restoring the nominal dimensions and preventing part disposal; with this aim, the technology has been investigated over flat surfaces [3] and even in challenging deposition conditions such as damaged edges [4]. The same process has been studied to the purpose of freeform fabrication, aiming to control the crystal orientation in metals and eliminating residual pores for superalloys [5]; indeed, DED is expected to have high potential in large-scale aerospace and automotive production [6].

At present, many needs must be addressed in this field: reducing the experimental trial-and-error approach to find the optimum domain for processing [7], providing feedback control [8], revealing the influence of the governing factors on heat transport [9]. Analytical methods have been developed [10], although a reliable description must include many complex concurrent physics. As a potential alternative, wide research activity has been devoted to simulation tools based on finite elements methods, and even to simplified 2-dimension approaches [11]. Pros and cons of the models available for DED have been addressed in depth and are comprehensively discussed in the literature [12]: in general, most of them rely on the available knowledge about simulation of other laser-assisted applications such as drilling [13], cutting [14], welding [15,16], surface hardening [17], ablation [18] and powder-bed fabrication [19]. Indeed, some common assumptions regarding the material properties and the heat source can be shared successfully among different models to address the main steps of laser heating and heat transport, irrespective of the technology involved [20].

**Figure 1:**
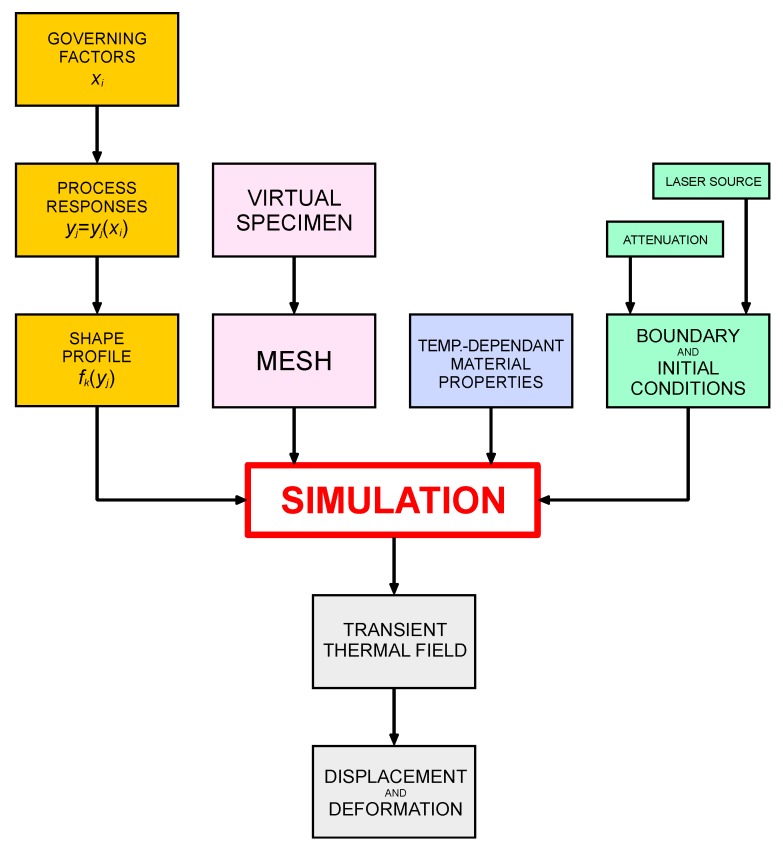
Logical flowchart of the global input to feed the simulation tool and compute the output.

Based on the current status of the literature, in order to properly discuss which items of the simulation of DED are required special focus and research, one may conveniently consider a flowchart scheme (Figure 1) representing the relevant steps to model this process. Each item is discussed in the following sections, thus highlighting the current research gaps in the field.

### 1.1. Size and Geometry

First, specific DED-related governing factors *x_i_* must be considered. In a lean model, only few of these factors are usually taken into account, although it has been pointed out that the outcome is affected by at least 12 main processing variables [21] and is highly sensitive to disturbances [22]. The levels of the processing factors decide the geometrical responses *y_j_* in terms of track width, height, depth and shape angles, via a dependence *y_j_*(*x_i_*) which is either empirical or theoretical; this law is available or can be found at a preliminary stage on a case-by-case basis. With respect to this subject, some effort has been made in the literature [23,24] to find a dependence of the size of the deposited metal on the processing factors (i.e., laser power, laser speed, feeding rate of powder) and the equipment (i.e., type, size and positioning of the feeding nozzle) in case of single-track deposition; and one of the main findings [25] is that the size of a clad bead can be easily shaped using power control. Nevertheless, for given width and height, a further step of investigation is required to find and validate the best interpolating shape function *f_k_* which is far from simple: many profiles (e.g., parabolic, circular and sinusoidal segments, semi-ellipses) have been proposed [26,27], although simplifications are usually made in the simulation-related literature, where a simple square profile has even been adopted [28]. The step is crucial because a known geometry of the deposited material over the substrate is required and must be fed to the simulation tool as a main input; indeed, direct modeling of the powder particles is not feasible. It is worth noting that any supposed shape function *f_k_* must be validated in advance, before being considered in the simulation; furthermore, the elected model for single deposition must be further extended to recursively predict the eventual profile of a larger coating formed by overlapping tracks [26], which is common practice in actual applications of maintenance and overhaul. With this approach, any governing factor can be involved implicitly via the shape function.

### 1.2. Space and Time Scale

Another major issue is setting the mesh size and the time step for the numeric solver, respectively. An adaptive approach is accepted in the available literature as the best solution for meshing [20,29], with finer elements where steeper gradients of properties are expected. As regarding the time step, it is worth noting that different timescales are generally involved. These range from milliseconds for the interaction time between the laser beam and the material, to minute for single-track deposition, to even hours of a global heat treatment during the overall manufacturing of a large part [30]. As a consequence, achieving a compromise on the time step is usually challenging.

### 1.3. Material Properties and Conditions

To complete the global input, additional tasks of setting the boundary condition of thermal exchange and feeding some temperature-dependent material properties must be addressed. These are simplified or neglected at all in the existing literature [10,31], at the discretion of the investigating authors and depending on the available capability of calculation. In this frame, even the implementation of an approximated distribution of laser heat generation may affect the outcome of the simulation: indeed, a conventional Gaussian beam is a common assumption, although not entirely fit for the purpose, since true lasers may deviate significantly from ideal distributions of irradiance [32]; a super-Gaussian distribution should be modelled, instead [20]. 

Eventually, on the subject of the interaction between the laser and the material, attenuation of the laser beam due to continuous powder injection must be considered [22], otherwise an overestimation of the simulated thermal field and a flawed prediction of the geometry may result [33]. Since the phenomenon is far from simple, some authors have focused specifically on the identification of a model for laser-powder coupling for DED [9]. On the other hand, with respect to modelling of higher-density laser-assisted processes, a key-hole is not produced in DED [34], as vaporization of metal is prevented, therefore plasma and related plume dynamics at near-infrared laser wavelength can be neglected [35].

### 1.4. Output of Modelling and Aim

The main eventual aim is computing the thermal history and solving the mechanical problem in turn. In general, the prediction of the transient temperature field is aimed since it directly affects residual stresses, microstructure, fatigue life and overall deformation of the parts [30,36]. 

Given the complex background as exposed before, a study is presented in this paper to offer a structured path for the simulation of DED, in terms of modelling input and validation tools. Namely, deposition of AA 2024 powder over homologous substrate is considered. The selection of the material is given grounds by its wide use in aerospace and automotive [37], even to manufacture high price sensitive parts which may require maintenance via DED [38]. 

The regression models for the geometric responses as a function of the governing input factors are computed in advance; then, the best shape function fitting the actual profile in single-deposition is investigated. The elected shape is hence shifted to cases of multi-track, multi-layer deposition with different overlapping ratios, to assess the effectiveness of the prediction of the overall geometry to feed the numerical solver. Therefore, the reliability of a simulation model, generated via COMSOL Multiphysics, is discussed to the purpose of extracting the extent of the fusion zone, as well as the deformation of the substrate, based on the thermal evolution of the metal, in single- and multi-track deposition. With respect to other similar attempts in the literature, special care is given to expressing the shape of the deposited metal as a function of the governing factors, implementing the properties of the base material as a function of the temperature and modelling the heat source according to a realistic distribution of energy. Moreover, a non-stationary problem is addressed, in order to extract any crucial information during the evolution of the process, such as the resulting displacement of a point of interest.

## 2. Materials and Methods 

**Figure 2:**
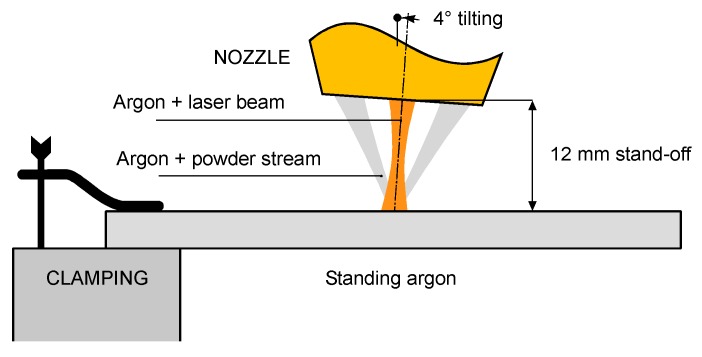
Scheme of the laser head with three-way feeding nozzle; components not to scale.

To validate both the analytical expression suggested for the responses of the process and the outcome of the numerical simulation, a number of experimental trials are referred here. These trials have been conducted using a laser deposition line [23] consisting of an Yb:YAG thin-disc laser source (Table 1), a feeder delivering the metal powder to the substrate and an industrial robot to move the laser head, equipped with a three-way feeding nozzle (Figure 2). Argon has been used to carry the powder and shield the metal to prevent oxidation, at flow rates of 3 and 10 L·min^−1^, respectively. The tip of the feeding nozzle has been moved to a stand-off distance of 12 mm, with a tilting angle of 4° to prevent possible damages due to back-reflections [39]. To increase the catching efficiency [22], a defocused beam has been used to produce a processing spot size of 3 mm.

The AA 2024 powder of nominal commercial chemical composition [37] is spherically shaped, its diameter ranges from 20 to 60 mm; the powder has been preliminary dried, in a furnace, at a temperature of 180 °C for a period of 2 h, aiming to ensure steady feeding.

Both in single- and multi-track depositions, 55 mm long paths have been set over 80 mm long, 60 mm wide, 10 mm thick plates. To check the effectiveness of the predictions, a custom-designed experimental plan has been arranged, in compliance with previous campaigns [23,40] and aimed to prevent defects, such as porosity which is a major issue in fusion of aluminium [38]; moreover, technical constraints of the equipment have been taken into account to limit the processing window.

Areal measurements of the deposited metal and the fusion zone have been obtained in the cross-section of single- and multi-track, multi-layer depositions: to this purpose, the samples have been cross-cut with respect to the direction of deposition, then mechanically grinded and polished to mirror finish, eventually chemically etched with a solution of nitric, hydrofluoric acid and water at room temperature [37]. The observation has been conducted by means of conventional optical microscopy.

## 3. Analytical Models for Size and Shape

### 3.1. Regression Models for the Geometric Responses

The first step towards simulation is the selection of a reliable regression model to express the size of the deposited metal in terms of width and height. It is known from the literature that simple linear models fail to be effective when wide processing domains are considered [23,41]; exponential laws are successful instead [42]. For *P*, *s* and *m* denoting laser power, scanning speed and feeding rate, respectively, and being *y* the generic geometric response, either width or height, these models state:

(1) $$y=k_{1}\cdot P^{\alpha }\cdot s^{\beta }\cdot m^{\gamma }+k_{2}$$

The exponents α, β and γ, as well as the calibration parameters *k*_1_ and *k*_2_ depend on the material and the laser equipment; in the literature, these values have been found for stainless steel processed by a diode laser [43] and titanium alloy Ti-6Al-4V processed by a Nd:YAG laser [44]. Thanks to a set of experimental trials (Table 2), the model of regression has been calibrated here at the 5% significance level for AA 2024, processed by an Yb:YAG laser (Table 3), for the purposes of this paper.

It is worth noting that the sign of each exponent is an indication for the effect of the corresponding factor on the response. Therefore, consistently with similar findings in the literature [38,45], both width and height decrease for increasing speed; on the other hand, they increase for increasing feeding. Moreover, increasing power yields an increase in width, in conjunction with a decrease in height, since powder is provided to a larger melting pool. As expected [41,42], width is only mildly affected by the feeding rate. 

The models are capable of replicating the fed data with average absolute accuracy of 7.4% for width, 9.8% for height; *R*-squared of 93.6 and 92.6% are offered, respectively. An accuracy of 5.9% and 6.3% resulted in predicting new results (Table 4), not included in the original database to train the model. Based on these findings, an expression of the shape profile as a function of the governing factors is viable. 

### 3.2. Geometry of the Deposited Metal

#### 3.2.1. Shape Functions

**Figure 3:**
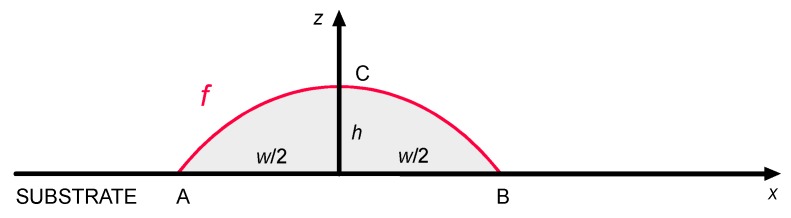
Shape function *f* for the deposited metal in the cross-section, single-track deposition.

Once a regression equation is available for the size of the deposited metal, a shape function *f* must be found to draw the geometry interpolating three given points A, B and C (Figure 3), based on the expected width *w* and height *h* resulting from Equation (1).

**Figure 4:**
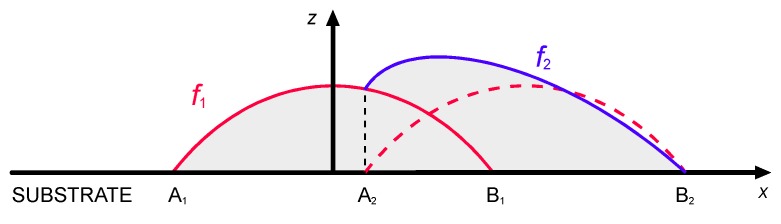
Shape functions *f_i_* for the deposited metal in the cross-section, overlapping deposition.

Then, the selected shape function must be used to implement multi-track processing with overlapping depositions, under the main assumption of mass conservation. Therefore, the amount of deposited material in any overlapping deposition is constant, in agreement with a successful approach proposed in the literature [27]. Namely, irrespective of the specific shape function *f*, mass balancing yields (Figure 4):

(2) $$\overset{B_{2}}{\underset{A_{2}}{\int }}f_{2}dx=\overset{B_{1}}{\underset{A_{1}}{\int }}f_{1}dx+\overset{B_{1}}{\underset{A_{2}}{\int }}f_{1}dx$$

As a consequence, the shape of the second adjacent track is determined by the shape of the first deposition. In general, in a series of *n* depositions in single-layer processing:

(3) $$\overset{B_{i}}{\underset{A_{i}}{\int }}f_{i}dx=\overset{B_{1}}{\underset{A_{1}}{\int }}f_{1}dx+\overset{B_{i-1}}{\underset{A_{i}}{\int }}f_{i-1}dx\mathrm{for}\;i=2,3,\ldots n$$

For a given condition of processing, it has been shown in the previous section that the width *w* is known as a function of the governing factors, therefore the locations of points *A_i_* and *B_i_* depend on the distance *d* between the axes of neighbouring tracks. To take account of this, the overlapping ratio *OR* is defined as:

(4) $$OR=\frac{w-d}{w}$$

As regarding multi-layer deposition, the previous layer is considered as the available substrate, then the process is iterated. Therefore, the resulting multi-track, multi-layer theoretical profile is built and fed to the simulation tool, recursively. Both in multi-track and multi-layer deposition, the catching efficiency and the mass of the deposited metal are expected to depend on the surface offered upon the previous action [22]; therefore, the hypothesis of constant deposited mass should be adjusted. Even the delay between two consecutive depositions, hence the residual heat, may still affect the following track due to a change in the surface tension. As a consequence, a dynamic evolution of the profile model should be used [10], based on adjusted catching efficiency and transient temperature field.

#### 3.2.2. Single Track Deposition

**Figure 5:**
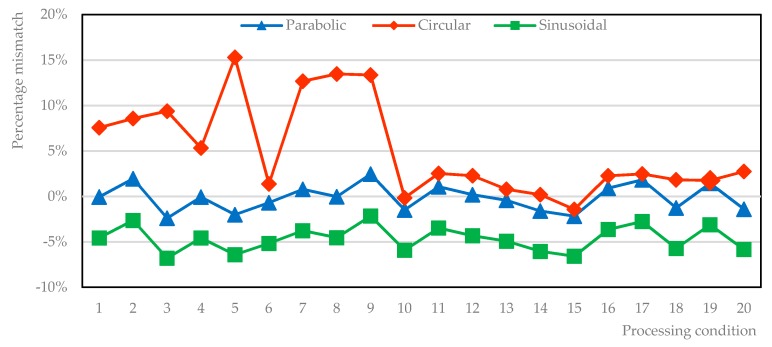
Percentage mismatch, with respect to the actual area of the deposited metal; approximation with semi-ellipse, resulting in wider mismatches, is omitted here.

The effectiveness of parabolic, circular and sinusoidal segments and semi-ellipses, as potential shape functions *f* for the deposited metal, is considered and tested here. A total of 20 operating conditions, resulting from the previous steps to compute (Table 2) and validate (Table 4) the regression equations are referred. Namely, actual width and height are considered to draw the interpolating profile, whose subtended area with respect to the reference line of the substrate (Figure 3) is compared to the actual areal response (Table 5, Figure 5). The approximation of a parabolic segment is effective in a measure of 1.2% on absolute average, whereas mismatches of 5.3% and 4.7% result in case of circular and sinusoidal segments. 

Moreover, the sinusoidal approximation yields to underestimation of the deposited metal; conversely, the circular approximation generally yields to overestimation and the approximation with semi-ellipses results in even wider mismatches, 135% on average.

Based on these findings, a parabolic shape function is selected to feed the simulation and will be implemented even in recursive approach according to Equation (3), to model multi-track depositions. Therefore, the profile *f* of the deposited metal is given as:

(5) $$f\left(w,h\right)=-\frac{4h}{w^{2}}x^{2}+h$$

Width and height, in turn, are expressed based on the equation of the regression models (1) and the corresponding coefficients (Table 3); as a result, the shape profile can be effectively scaled as a function of the operating conditions.

#### 3.2.3. Multi-track, Multi-layer Deposition

**Figure 6:**
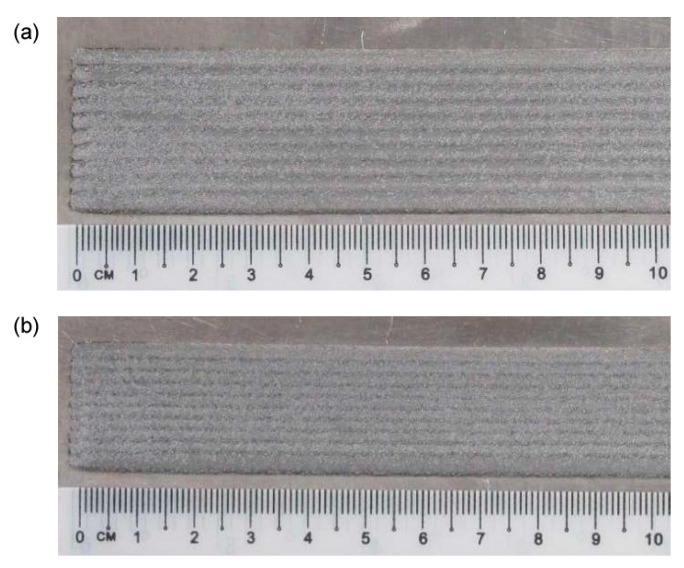
Surface aspects, multi-track, single-layer deposition: (**a**) 33% *OR*; (**b**) 50% *OR*.

**Figure 7:**
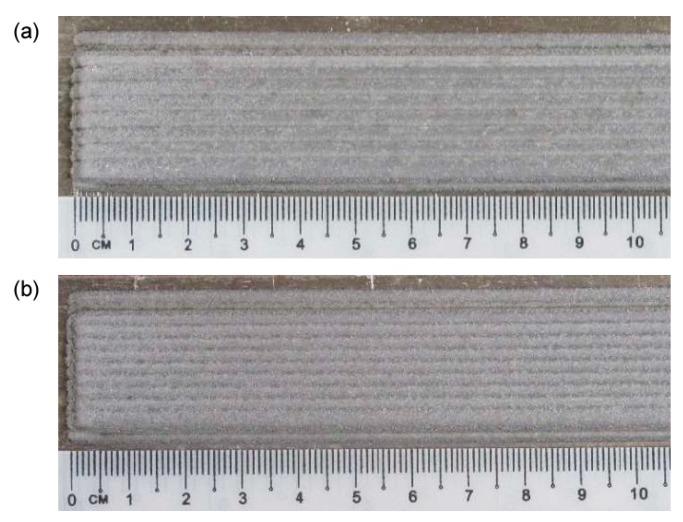
Surface aspects, multi-track, 2-layer deposition: (**a**) 33% *OR*; (**b**) 50% *OR*.

**Figure 8:**
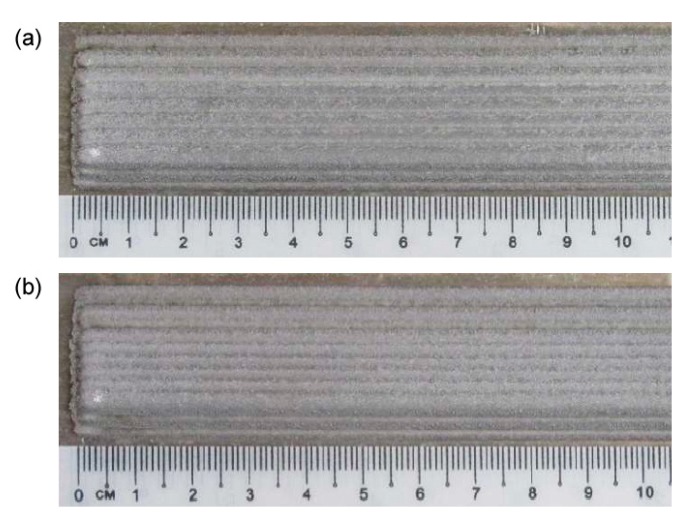
Surface aspects, multi-track, 3-layer deposition: (**a**) 33% *OR*; (**b**) 50% *OR*.

To validate the recursive approach to draw the profile of the deposited metal in multi-track deposition, a single condition (2.0 kW power, 500 mm·min^−1^ speed and 7 g·min^−1^ feed rate) has been selected and taken, being this set-up beneficial to the specific purpose of minimum dilution (i.e., reduced affection of the substrate with respect to effective deposited metal). As regarding the deposition strategy, 33 and 50% *OR* have been set: single-, 2- and 3-layer depositions have been performed (Figure 6, Figure 7 and Figure 8). 

**Figure 9:**
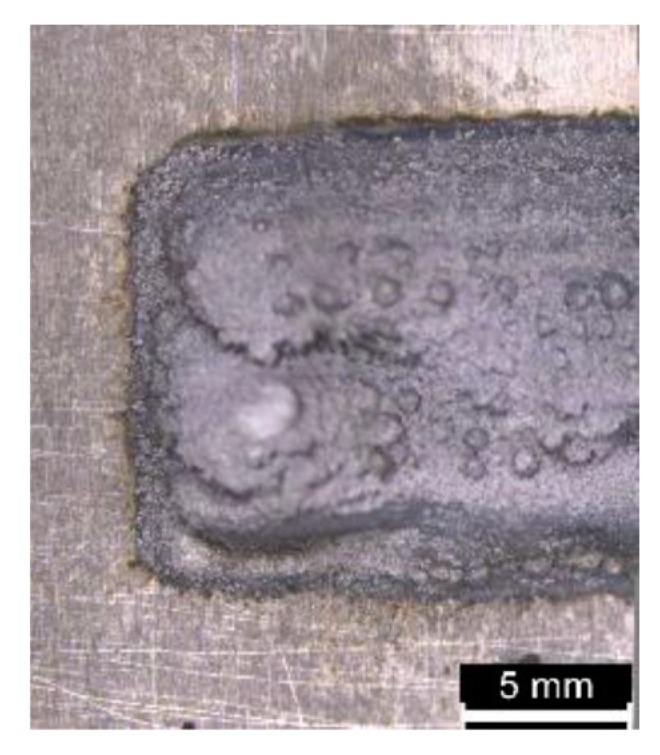
Detail of surface irregularities of the first layer for 75% *OR*.

The condition with *OR* exceeding 50% has been discarded from further investigations because it resulted in irregular surface of the first layer hindering any possible subsequent deposition of the next one (Figure 9). 

**Figure 10:**
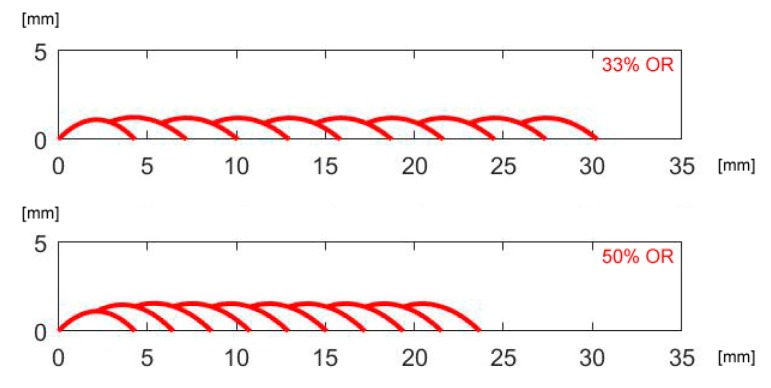
Multi-track, single-layer deposition for given *OR*; theoretical profiles.

**Figure 11:**
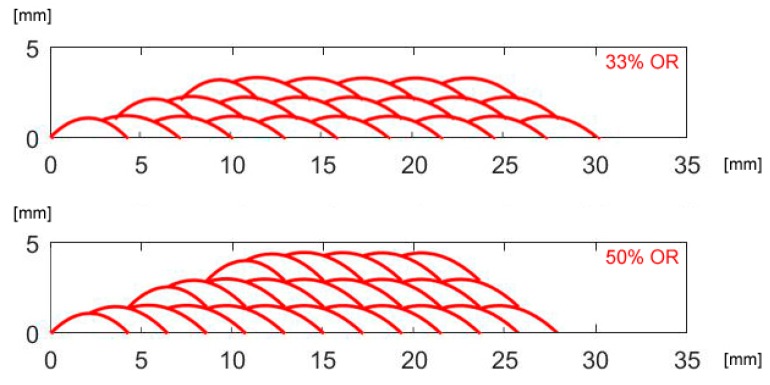
Multi-track, multi-layer deposition for given *OR*; theoretical profiles.

According to the geometrical prediction of multi-track deposition (Figure 10), the track height (Table 6) is constant once a given number of tracks, depending on the *OR*, are laid. As a consequence, when moving to multi-layer deposition (Figure 11), a side offset must be set for each layer with respect to the previous layer to offer a regular substrate to the laser beam and the delivering nozzle; the prediction of the mean height of each layer has been used, to shift the laser head upwards, i.e., to adjust the stand-off of the laser head when programming the deposition path. 

**Figure 12:**
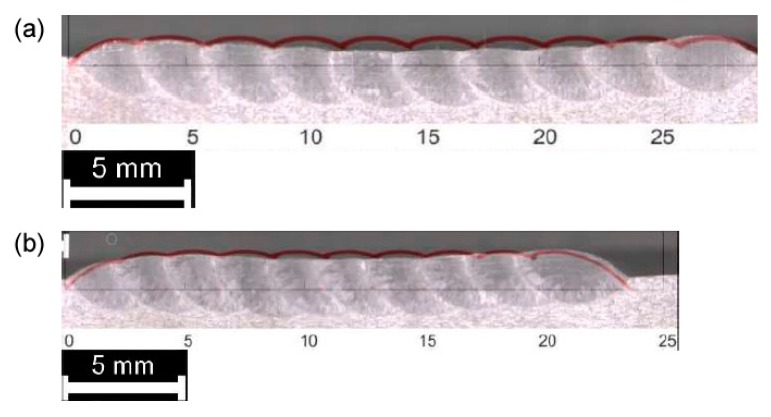
Comparison between theoretical (red, superimposed) and actual profile, multi-track, single-layer deposition: (**a**) 33% *OR*; (**b**) 50% *OR*.

**Figure 13:**
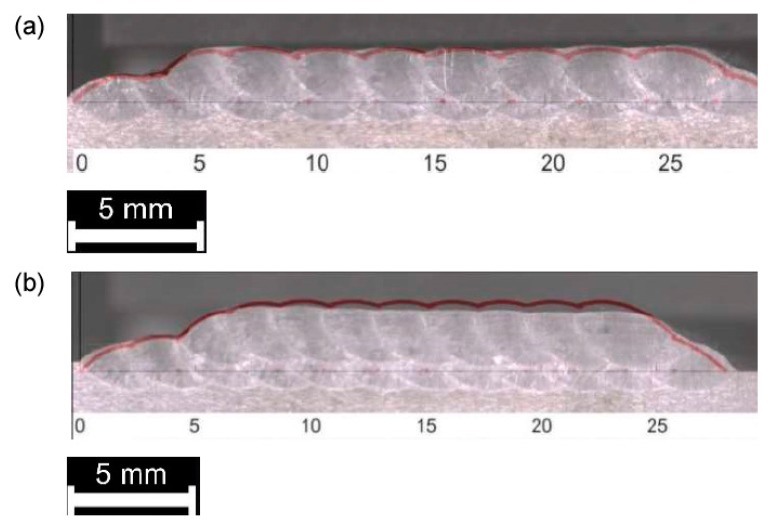
Comparison between theoretical (red, superimposed) and actual profile, multi-track, 2-layer deposition: (**a**) 33% *OR*; (**b**) 50% *OR*.

**Figure 14:**
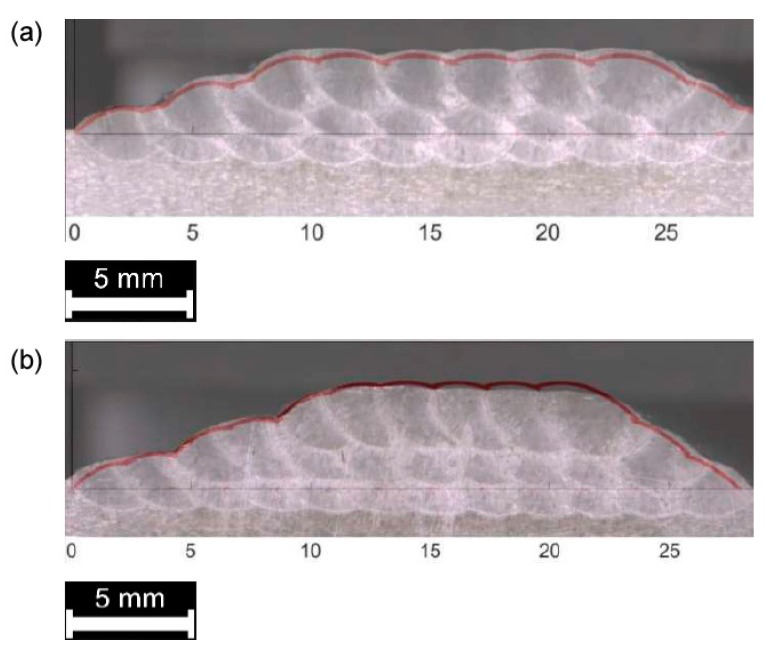
Comparison between theoretical (red, superimposed) and actual profile, multi-track, 3-layer deposition: (**a**) 33% *OR*; (**b**) 50% *OR*.

As for single deposition, the theoretical multi-track and multi-layer profile must be compared to the actual cross-section for each processing condition (Figure 12, Figure 13 and Figure 14). Again, the theoretical subtended area is referred to assess the effectiveness of the approximation with respect to the total deposited metal (Table 7) and the surface of the substrate is considered as the reference line for comparison.

It is worth noting that the subtended area of the first layer is the same, irrespective of *OR*, as a consequence of mass balancing in Equation (3); for the subtended area of the other layers, instead, a dependence on the *OR* is in place, because different heights are produced (Table 6). The actual area is matched in measure of 5%, on absolute average; higher mismatches, due to severe thermal cycles and buckling of the substrate are possible when thinner plates are used. 

The valuable milestone of this investigation is that the analytical description of the deposited metal is always possible, in single-, multi-track and multi-layer deposition, to draw the required geometrical input for simulation.

## 4. Simulation Tool

### 4.1. Virtual Specimen and Mesh

**Figure 15:**
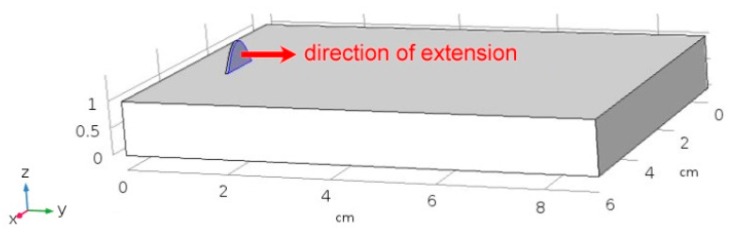
Implementation of a deformed geometry to result in the addition of metal.

A virtual specimen of the same size of the actual plate for the experimental trials must be modelled. To simulate the addition of metal during DED, a domain of increasing length is created (Figure 15) by means of extending a starting reference shape function along the laser path (i.e., in the *y* direction). To this purpose, the method of the deformed geometry is available in COMSOL: the processing speed is set as deforming speed to extend a finite, 0.5 mm thick slice to an overall length of the laser path.

**Figure 16:**
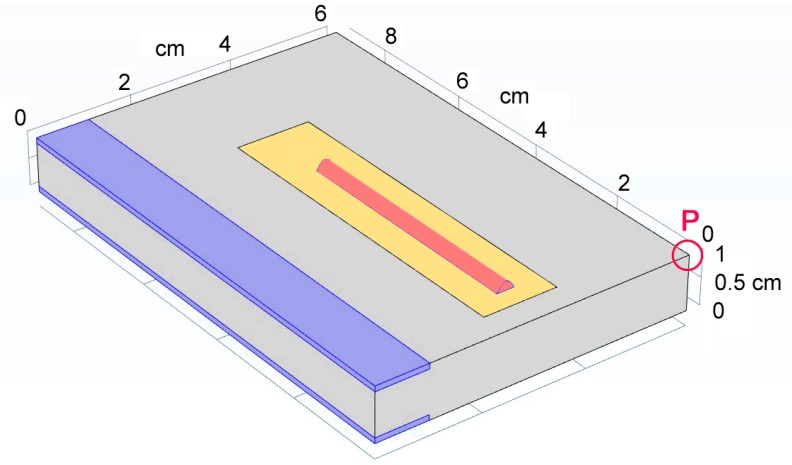
Domains of interest: deposited metal (red, *D*_1_), central slot (yellow, *D*_2_), remaining parent metal (grey, *D*_3_) and side slices for mechanical constraints (blue, D_4_); point of interest P to monitor the resulting strain of the part.

Four domains of interest *D* over the virtual specimen (Figure 16) are considered and are given thermal and mechanical continuity: the deposited metal (domain 1, *D*_1_); a slot, 10 mm wide, 0.4 mm deep, to apply the laser path (domain 2, *D*_2_); the remaining parent metal (domain 3, *D*_3_); two side slices, 1 mm thick, for mechanical constraints (domain 4, *D*_4_). A point of interest *P* is even selected at this stage, to the purpose of further investigation about combined thermal and mechanical strain of the component.

**Figure 17:**
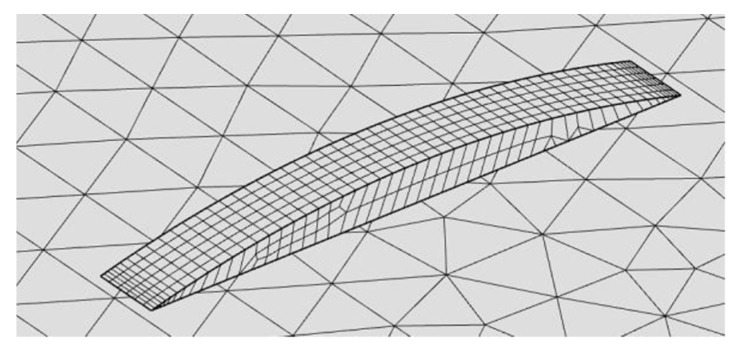
Mesh detail over the deposited metal; quad faces of 0.045 mm average edge size.

**Figure 18:**
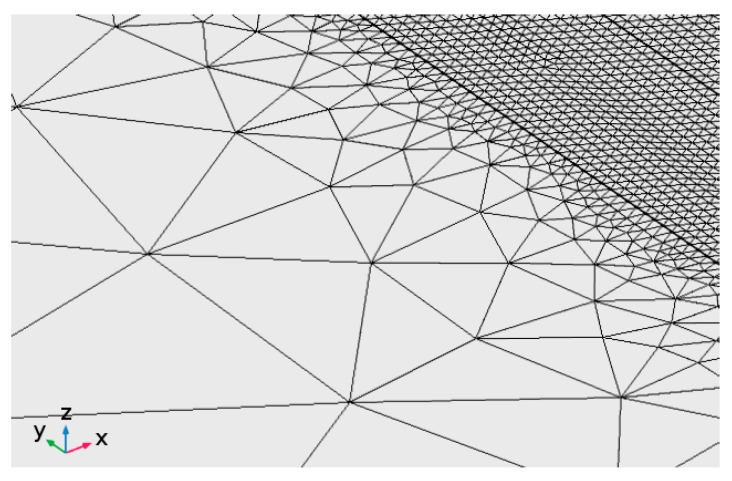
Mesh detail at the interface between the slot and the remaining parent metal; triangle faces in the range from 0.20 to 10 mm edge size.

To improve the consistency of the simulation, an adaptive mesh is appointed: as a general rule, finer elements are given to the deposited metal and along the laser path to properly take account of a focused beam and address steeper thermal gradients; coarser elements are given at the edges of the plate instead, aiming to diminish the computational effort. Moreover, to prevent excessive distortion of the mesh due to the extension of the deposited metal during simulation, quad faces with 0.045 mm average edge size are preferred for *D*_1_ (Figure 17); triangle faces, ranging from 0.20 mm in size within *D*_2_ to 10 mm across *D*_3_ and *D*_4_ (Figure 18) are generated.

### 4.2. Material Properties

The typical composition of the alloy for wrought products [37] is chosen for the parent metal. *Solidus* and *liquidus* temperature of 775 and 911 K, respectively, are known. Moreover, based on the available literature, the dependence on temperature is addressed for density [46], heat capacity [47,48], conductivity [48] and reflectivity [20] at the Yb:YAG wavelength; these are crucial to address the transient stage of processing.

In the solidification range between *liquidus* and *solidus* temperature, a general rule of mixtures [20] is implemented; therefore, depending on the solid volume fraction, the material properties are automatically updated by the solver in a two-phase model. Where required, to compute the radiative losses as discussed in the relevant section, a constant emissivity of 0.11 is set, upon experimental testing the emission of the parent metal using an IR camera, although a law for the dependence of the emissivity on the temperature field is strongly required for future adjustments of the simulation tool.

### 4.3. Heat generation

**Figure 19:**
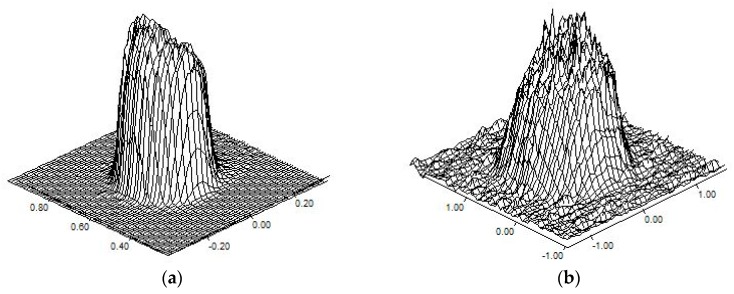
Distribution of transverse optical intensity, for 2.0 kW operating power, at (**a**) focus and (**b**) 18 mm defocused location required for the aimed processing diameter; pictures are not to scale between them.

Aiming to a reliable simulation of the heat transfer, the description of laser heat generation *Q*(*r*) as a function of the radial distance *r* from the axis of propagation is based on the assumption of a super-Gaussian profile distribution of irradiance [32], detected via preliminary experimental acquisitions using a beam profiler (Figure 19).

A smoothed flat-top profile is in place and a transverse optical intensity of order 20 has been implemented at the location of processing. Therefore, for *Q*_0_ denoting the peak intensity and *w*_0_ the beam radius over the incident surface, the laser heat generation is:

(6) $$Q\left(r\right)=Q_{0}\exp\left[-2{\left(\frac{r}{w_{0}}\right)}^{20}\right]$$

The effectiveness of this assumption to generate a reliable thermal response has been validated separately [20], when simulating mere laser heating of the same metal alloy as a reference scheme to model further laser processes. The peak intensity can be computed analytically and, based on the definition of irradiance, it is a function of the order of the distribution [49]. In this case:

(7) $$Q_{0}=1.1\frac{P}{\pi w_{0}^{2}}$$

where *P* is the operating power. Eventually, a moving heat source is set, with *x*_0_ and *y*_0_ denoting the starting point of the laser path, *s* the scanning speed along the *x*-direction and *t* the time:

(8) $$Q\left(x,y\right)=1.1\frac{P}{\pi w_{0}^{2}}\exp\left[-\frac{2\left[{\left(s\;t-x_{0}\right)}^{20}+{\left(y-y_{0}\right)}^{20}\right]}{w_{0}^{20}}\right]$$

Therefore, power and scanning speed are directly involved in the simulation via an explicit law. To take account of the feedstock, a direct simulation of the powder flow is not feasible instead. Nevertheless, since the operating irradiance is attenuated by powder injection, one may expect the reduction is proportional to the projected area of the in-flight particles within the laser beam [22]. Namely, for coaxial feeding of metal powder to a stand-off distance in the order of 10 mm, a radial symmetrical attenuation in a measure of 10% with respect to the theoretical heat source has been suggested and proved [27]. Reflected and emitted radiations are further deducted from the heat generation.

### 4.4. Boundary and Initial Conditions

Boundary and initial conditions apply for each domain. At first, within the operating window of processing and considering the characteristic lengths in the problem, the influence of the carrier gas on the velocity of the particle can be ignored; even collisions among the powder particles have minor probability of occurrence [9]. Moreover, based on time scale analysis [22,50], powder is considered to melt instantaneously within the melting pool, thanks to rapid heat transfer; therefore, the deposited metal defining *D*_1_ is activated in the simulation at the *solidus* temperature which is taken as a constant at the moving solidification front during the extrusion. Initial temperature for both *D*_1_ and *D*_2_ is assumed as room temperature, instead; then, heat flow according to Equation (8) entering *D*_2_ is set and ruled by a virtual binary switcher depending on the scanning speed.

Convection in argon is assumed over substrate and deposited metal, with constant coefficient of heat convection of 10 W·m^−2^·K^−1^ [47,48]; standing argon, hence negligible convection, is assumed under the plate.

Convection and radiation losses apply even during extrusion. As regarding *D*_2_: the generation of heat due to laser irradiation is provided along the path according to Equation (8) and phase change is implemented; again, convection and radiation losses apply at the upper surface of the substrate; a condition of thermal continuity has been given given with *D*_1_ at their shared surface. As regarding *D*_3_: each surface is affected by convection and radiation losses, but thermal and mechanical continuity must be assumed with respect to *D*_2_ at their shared surface.

As regarding the mechanical constraints, clamping of the plate is simulated by uniformly distributed loading of 5 kPa over *D*_4_ under the conditions of rigid motion suppression and mechanical continuity with the remaining parent metal.

## 5. Results and Discussion

### 5.1. Simulation of Single Deposition

Numerical simulations have been run and compared to the actual outcome, for a given flow rate of 3 g·min^−1^ over a wide domain of travelling speeds, spanning from 180 to 1800 mm·min^−1^, with laser power of 2.5 and 3.0 kW. This approach has been taken aiming to validate the reliability of the COMSOL solver in a broader window, over significantly different conditions in terms of thermal load and levels of the governing factors.

**Figure 20:**
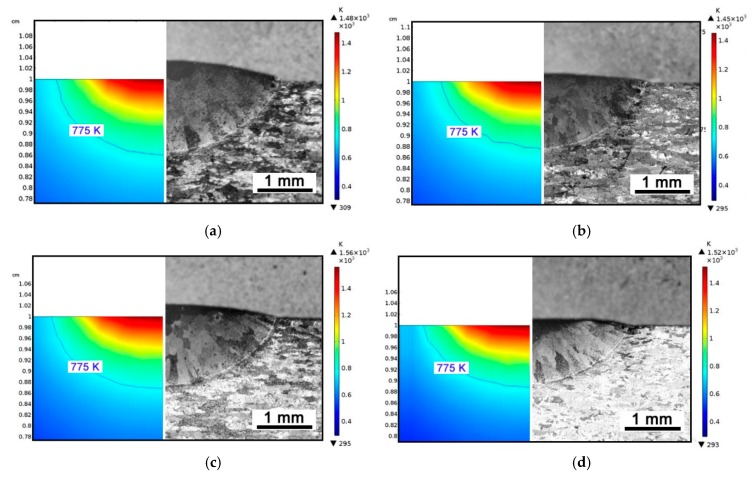
Comparison between simulated size (left side of each image) and actual size (right side of each image) of the fusion zone for given feeding rate of 3 g·min^−1^. Contour lines are drawn one time-step fraction before metal addition. (**a**) Power 2.5 kW; travelling speed 450 mm·min^−1^; (**b**) Power 2.5 kW; travelling speed 900 mm·min^−1^; (**c**) Power 3.0 kW; travelling speed 900 mm·min^−1^; (**d**) Power 3.0 kW; travelling speed 1800 mm·min^−1^.

Since the main eventual aim of simulation is computing the thermal history ruling the mechanical problem in turn, validation of the temperature profile over the parent metal is crucial: the size of the simulated fusion zone in the cross-section has been referred as indirect measure of temperature for validation. Namely, a transverse plane (i.e., in the *xz* plane) at half-length of the virtual specimen has been considered with respect to the travelling direction *y*, then thermal contour lines have been drawn (Figure 20, left half). Indeed, since 775 K is the *solidus* temperature, one may expect that fusion is experienced by any point above this limit. Therefore, width and depth of the fused metal (i.e., in the aggregated domains *D*_2_ and *D*_3_) can be inferred. For each given processing condition, these responses have been compared to the geometry (Figure 20, right half; Table 8) in the actual cross-section at half-length of the real specimen; the comparison has been conducted one time-step fraction before metal addition, i.e., before activation of the material in the corresponding cross-section. An agreement in average measure of 8.5% for width and 5.0% for depth, absolute, has been found. When extracting information from the simulation, different threshold temperatures could be set to validate even the extent of the heat-affected zone.

**Figure 21:**
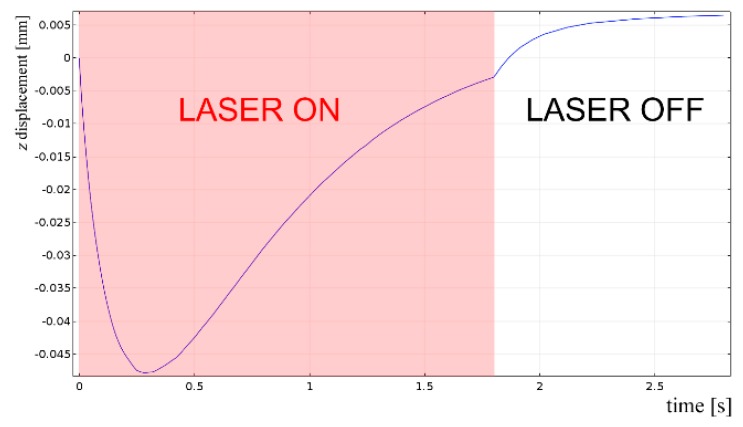
Displacement of the point of interest as a function of time; single deposition, 3.0 kW power, 1800 mm·min^−1^ speed and 3 g·min^−1^ feeding.

Interestingly, the simulation is helpful in predicting additional information which are crucial in practice, when repairing by DED must be performed over a damaged component and its deformation must be prevented. To this purpose, the displacement of the point of interest (Figure 16) in the direction of the propagation of the laser beam has been monitored during the virtual deposition and discussed: the trend (Figure 21) is ruled by expansion of the plate at laser switch-on, then contraction as the laser beam moves forward. The same trend has been found for the other simulated conditions, although the extent of the displacement is clearly scaled by the levels of the processing factors (Table 9): interestingly, a linear trend of the displacement can be inferred as a function of the thermal input (i.e., the power to speed ratio).

Moreover, thanks to focused energy delivering, the resulting displacement is three order of magnitude lower than the plate thickness, although is expected to increase in multiple depositions. Different trend and extent would result from different conditions of clamping.

### 5.2. Simulation of Multi-Track Deposition

**Figure 22:**
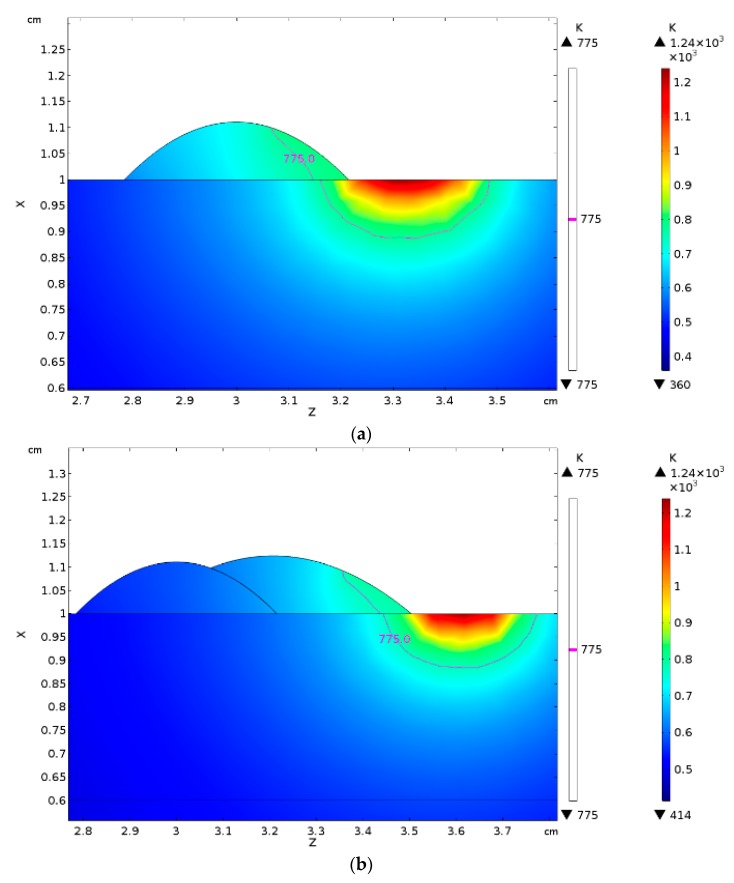
Multiple deposition: thermal field one time-step fraction before metal addition. (**a**) during second deposition; (**b**) during third deposition.

**Figure 23:**
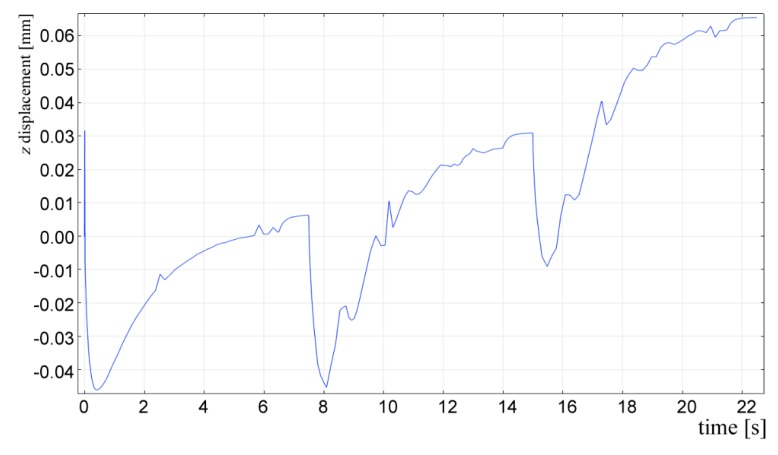
Displacement of the point of interest as a function of time; 3-track consecutive deposition strategy with no time delay.

Based on convincing validations of shape and temperature in single deposition, one may run the simulation for different, more complex cases. As an example, a case study of 3-track deposition is presented. The shape of the deposited metal has been computed according to Equation (3) for a given condition of 2.0 kW power, 500 mm·min^−1^ scanning speed, 3 g·min^−1^ feeding and 33% OR. Boundary and initial conditions apply as for the domain of the deposited metal in single deposition. No time delay has been set between consecutive depositions, which is consistent with real processing where DED is conducted by industrial robots. The chromatic continuity (Figure 22) along substrate and tracks is worth noting, being this a clear evidence of thermal continuity in different domains. As expected, the trend of displacement of the point of interest (Figure 23) is the result of repeated periods of expansion and contraction during processing, therefore the overall value is increased with respect to single deposition.

## 6. Conclusions

In this paper, a study has been presented to build a proper simulation tool for laser-assisted directed energy deposition of aluminum powder. The driving idea is to show that effective prediction and evolution of temperature and mechanical strain is possible in non-stationary models. Namely, one may conveniently change the levels of the governing factors to manage the response and possibly find an optimum condition of processing, to the specific purpose of the final application.

It is worth noting that a simulation implementing explicit dependences on any governing factor is not viable; nevertheless, a milestone has been set in this study since one may involve any additional factor, such as the feeding rate, by indirectly computing its effect on the size of the shape function. With this approach, grounds are given to take account of any phenomenon whose simulation is unfeasible.

- More specifically, the main findings of the research can be divided in two groups and listed as follows. At first, as regarding the geometry of the deposited metal:
- a parabolic segment is an effective approximation of the shape of the deposited metal in the cross-section;
- this can be given as a function of width and height, which are adequately modelled by exponential laws, whose parameters have been computed here for AA 2024 processed with Yb:YAG laser;
- a recursive method involving mass balance equations is successful to predict the geometry in multi-track, multi-layer deposition.

As regarding the simulation tool, an effort has been made to take account of many items which are often simplified in other similar approaches reported in the literature, depending on the available computational capability. The implementation of a proper shape is among them; in addition, this paper addressed a realistic description of the distribution of irradiance of the heat source and the dependence of the material properties on the temperature. As a result, the suggested model:

- is able to match the size of the fusion zone, as indirect validation of the predicted temperature field, in average measure of 6.8%;
- is capable of extracting additional mechanical information about displacement, whose trend is consistent with the dynamics of the process and is approximately linear function of the thermal input.

Nevertheless, reliable modelling is still challenging: indeed, a huge computational effort is required to simulate multi-layer deposition; some simplifications of meshing, boundary conditions and properties may ease the challenge, although this could seriously impact on the accuracy of the results, to an extent to be investigated in future works. Even the dependence of the emissivity on temperature should be found and implemented to the numerical solver.

## Figures and Tables

**Table 1:** Main features of the Yb:YAG thin disc laser source.

Parameter	Value
Maximum output power	4 kW
Operating nominal wavelength	1030 nm
Beam Parameter Product	8.0 mm × mrad
Processing diameter	3 mm

**Table 2:** Actual and simulated responses with absolute percentage mismatch for each processing condition, to calibrate the regression model.

Processing Conditions			Width of the Deposited Metal			Height of the Deposited Metal		
Power (kW)	Speed (mm·min^−1^)	Feeding (g·min^−1^)	Actual (mm)	Predicted (mm)	Mismatch (%)	Actual (mm)	Predicted (mm)	Mismatch (%)
1.2	400	4	3.09	2.91	5.9	0.98	1.08	10.1
1.5	400	8	3.49	3.63	3.9	1.98	1.73	12.4
1.5	400	10	4.30	3.68	14.3	2.14	2.13	0.6
2.0	200	10	5.28	6.38	20.8	2.11	2.41	14.0
2.0	400	8	4.07	4.52	11.1	1.75	1.45	17.0
2.0	500	8	3.94	4.07	3.2	1.49	1.32	11.3
2.5	200	5	8.05	7.20	10.6	1.05	1.12	7.1
3.0	150	5	8.60	9.46	9.9	1.16	1.13	2.2
3.0	200	5	8.05	8.26	2.5	1.00	1.01	1.0
3.0	200	7	8.80	8.46	3.9	1.34	1.35	0.8
3.0	200	4	8.08	8.14	0.7	0.79	0.84	6.3
3.0	200	6	9.22	8.37	9.2	1.20	1.18	1.6
3.0	200	3	7.50	7.97	6.3	0.67	0.67	0.2
3.0	200	10	9.30	8.67	6.8	1.86	1.86	3.3
3.0	600	3	4.67	4.74	1.5	0.29	0.46	58.5

**Table 3:** Exponents and calibration parameters of regression for width and height; measuring units of the factors as deposited in Table 2.

Response	*k*_1_	*k*_2_	α	β	γ
Width	38.729	−0.110	0.745	−0.464	0.069
Height	4.529	0.154	−0.686	−0.474	0.994

**Table 4:** Actual and simulated responses with absolute percentage mismatch for each processing condition, to validate the regression model.

Processing Conditions			Width of the Deposited Metal			Height of the Deposited Metal		
Power (kW)	Speed (mm·min^−1^)	Feeding (g·min^−1^)	Actual (mm)	Predicted (mm)	Mismatch (%)	Actual (mm)	Predicted (mm)	Mismatch (%)
1.5	400	4	3.71	3.45	6.9	1.08	0.95	12.3
1.5	400	6	3.61	3.55	1.6	1.46	1.34	8.1
2.0	200	3	5.24	5.87	11.9	0.85	0.83	1.9
3.0	150	7	9.41	9.68	2.9	1.53	1.52	0.4
3.0	600	10	5.50	5.19	6.2	1.28	1.17	8.8

**Table 5:** Comparison among actual and subtended area of the deposited metal in single-track deposition for different shape: parabolic (P), circular (C), sinusoidal (S) and semi-ellipses (E).

Processing Conditions			Area of the Deposited Metal (mm^2^)								
Power (kW)	Speed (mm·min^−1^)	Feeding (g·min^−1^)	Actual	P	Mismatch	C	Mismatch	S	Mismatch	E	Mismatch
1.2	400	4	2.02	2.019	−0.1%	2.173	7.6%	1.928	−4.6%	4.760	135.5%
1.5	400	4	2.62	2.671	2.0%	2.844	8.6%	2.551	−2.6%	6.290	140.2%
1.5	400	6	3.60	3.514	−2.4%	3.938	9.4%	3.355	−6.8%	8.280	130.0%
1.5	400	8	4.61	4.607	−0.1%	4.856	5.3%	4.399	−4.6%	10.85	135.5%
1.5	400	10	6.26	6.135	−2.0%	7.218	15.3%	5.858	−6.4%	14.45	130.9%
2.0	200	3	2.99	2.969	−0.7%	3.031	1.4%	2.836	−5.2%	7.000	134.0%
2.0	200	10	7.37	7.427	0.8%	8.304	12.7%	7.092	−3.8%	17.50	137.4%
2.0	400	8	4.75	4.748	0.0%	5.390	13.5%	4.534	−4.5%	11.19	135.5%
2.0	500	8	3.82	3.914	2.5%	4.330	13.4%	3.737	−2.2%	9.220	141.4%
2.5	200	5	5.72	5.635	−1.5%	5.711	−0.2%	5.381	−5.9%	13.28	132.1%
3.0	150	5	6.58	6.651	1.1%	6.746	2.5%	6.351	−3.5%	15.67	138.1%
3.0	150	7	9.58	9.598	0.2%	9.798	2.3%	9.166	−4.3%	22.62	136.1%
3.0	200	5	5.39	5.367	−0.4%	5.432	0.8%	5.125	−4.9%	12.64	134.6%
3.0	200	7	7.99	7.861	−1.6%	8.005	0.2%	7.507	−6.0%	18.52	131.8%
3.0	200	4	4.35	4.255	−2.2%	4.288	−1.4%	4.064	−6.6%	10.03	130.5%
3.0	200	6	7.31	7.376	0.9%	7.475	2.3%	7.044	−3.6%	17.38	137.7%
3.0	200	3	3.29	3.350	1.8%	3.371	2.5%	3.199	−2.8%	7.89	139.9%
3.0	200	10	11.68	11.532	−1.3%	11.893	1.8%	11.012	−5.7%	27.17	132.6%
3.0	600	3	0.89	0.903	1.4%	0.906	1.8%	0.862	−3.1%	2.13	139.0%
3.0	600	10	4.76	4.693	−1.4%	4.891	2.7%	4.482	−5.8%	11.06	132.3%

**Table 6:** Prediction of track height for a given layer as a function of the *OR*.

Track	1	2	3	4	5	6	7	… *n*
Track height (mm) with 33% *OR*	1.11	1.24	1.21	1.21	1.21	1.21	1.21	1.21
Track height (mm) with 50% *OR*	1.11	1.48	1.56	1.55	1.54	1.54	1.54	1.54

**Table 7:** Comparison between actual and theoretical area of the deposited metal for given *OR* in multi-layer deposition.

OR	Number of Layers	Actual Area of Deposited Metal (mm^2^)	Subtended Theoretical Area (mm^2^)	Mismatch
33%	1	36.47	31.89	13%
33%	2	61.24	57.40	6%
33%	3	78.03	76.53	2%
50%	1	30.05	31.89	-6%
50%	2	67.76	66.97	1%
50%	3	82.44	86.10	-4%

**Table 8:** Actual vs. predicted size of the fusion zone in single track, for given feeding rate of 3 g·min^−1^.

Power (kW)	Speed (mm·min^−1^)	Width of Fusion Zone (mm)			Depth of Fusion Zone (mm)		
		Actual	Simulated	Difference	Actual	Simulated	Difference
2.5	450	4.29	4.00	−7.3%	1.30	1.35	+3.7%
2.5	900	4.08	3.60	−11.8%	1.22	1.20	−1.6%
3.0	900	4.23	3.80	−10.2%	1.29	1.30	+7.7%
3.0	1800	3.88	3.63	−4.5%	1.05	1.10	+6.8%

**Table 9:** Displacement of the point of interest as a function of delivered thermal input, for given feeding rate of 3 g·min^−1^.

Power (kW)	Speed (mm·min^−1^)	Thermal Input (J·mm^−1^)	*z*-displacement (mm)
2.5	450	333	0.016
3.0	900	200	0.010
2.5	900	166	0.007
3.0	1800	100	0.005
